# Functional Segregation‐Integration Preference Configures the Cognitive Decline Against Cerebral Small Vessel Disease: An MRI Study

**DOI:** 10.1111/cns.70162

**Published:** 2024-12-17

**Authors:** Wentao Hu, Yao Wang, Zhenhui Xie, Mianxin Liu, Xu Han, Ying Hu, Xingrui Wang, Yongming Dai, Qun Xu, Yan Zhou

**Affiliations:** ^1^ Department of Radiology, Renji Hospital, School of Medicine Shanghai Jiao Tong University Shanghai China; ^2^ Shanghai Artificial Intelligence Laboratory Shanghai China; ^3^ School of Biomedical Engineering & State Key Laboratory of Advanced Medical Materials and Devices ShanghaiTech University Shanghai China; ^4^ Department of Neurology, Renji Hospital, School of Medicine Shanghai Jiao Tong University Shanghai China; ^5^ Renji‐UNSW CHeBA Neurocognitive Center, Renji Hospital, School of Medicine Shanghai Jiao Tong University Shanghai China; ^6^ Department of Health Manage Center, Renji Hospital, School of Medicine Shanghai Jiao Tong University Shanghai China

**Keywords:** cerebral small vessel disease, cognitive outcome, integration, resting‐state functional MRI, segregation

## Abstract

**Introduction:**

Cerebral small vessel disease (CSVD) is highly prevalent in elder individuals, and its variable cognitive outcomes indicate some cognitive reserve mechanisms. Contribution from functional network features is still unclear. Here we explore how functional segregation‐integration preference influences the cognitive changes against CSVD.

**Materials and Methods:**

A total of, 271 CSVD patients were included, all underwent MRI scans including routine and resting‐state functional MRI (rs‐fMRI). Hierarchical balance index (*H*_*B*_) was obtained from the rs‐fMRI connectivity using eigenmode‐based approach. Individuals were classified into segregated and integrated groups according to negative and positive *H*_*B*_. A composite CSVD lesion score was calculated from imaging findings. Global and five specific cognitive functions were assessed.

**Results:**

Hierarchical regression analysis revealed negative contribution from lesion load to global and all cognitive domains (*β* = −0.22~−0.35, ∆*R* ^2^ = 0.046~0.112, all *p* < 0.001). Inclusion of *H*_*B*_ did not show significant contribution (all *p* > 0.05), but interaction between *H*_*B*_ and lesion score was significantly associated with global (*β* = −0.27, ∆*R* ^2^ = 0.013, *p* = 0.034) and execution score (*β* = −0.34, ∆*R* ^2^ = 0.023, *p* = 0.002). Integrated patients show significant better global cognitive (23.9 ± 3.9 vs. 25.5 ± 3.1, *p* = 0.044) and executive ability (0.235 ± 0.678 vs. 0.535 ± 0.688, *p* = 0.049) at mild damage stage, visuospatial (−0.001 ± 0.804 vs. 0.379 ± 0.249, *p* = 0.034) and language ability (−0.133 ± 0.849 vs. 0.218 ± 0.704, *p* = 0.037) at moderate damage stage. Cross‐overs of cognitive scores were observed. Significant better execution (−0.277 ± 0.717 vs. −0.675 ± 0.883, *p* = 0.027) was found in severe damage stage for segregated patients.

**Conclusion:**

Thus, we concluded that integrated network contributes to cognitive resilience in mild and moderate but not in severe damage stages.

## Graphical abstract

This study emphasizes the impact of functional segregation‐integration preference on the cognitive outcome of cerebral small vessel disease (CSVD). Compared to segregated resting‐state network, integrated one was found to contribute to cognitive resilience against mild and moderate damage from CSVD. Cognitive cross‐overs exist for several domains before severe damage stages.

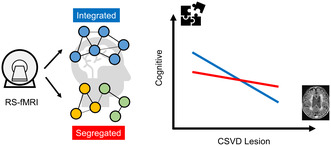

## 1 Introduction

Cerebral small vessel disease (CSVD) is one of the most prevalent age‐related processes encountered in clinical practice [1, 2]. It pathologically affects the small blood vessels including arterioles, venules, and capillaries [2, 3], leading to ischemic or hemorrhagic lesions, manifested with MRI findings including white matter hyperintensities (WMH), lacunar infarcts (LI), cerebral microbleeds (CMB), and gray matter atrophy [4, 5]. As an important consequence of CSVD, cognitive impairment evolves and contributes to 40%–50% of all dementias [2, 6]. Nevertheless, the extent of cognitive impairment varies a lot. While some CSVD patients with large WMH lesions experience only mild or even no cognitive symptoms, others may quickly evolve to vascular dementia [7]. Some with mild cognitive impairment may revert to normal level over time while most patients do not [8, 9]. As such, various cognitive outcome challenges the clinical management of CSVD.

Cognitive variability implies the exist of underlying mechanisms preserving or compensating the brain functions [10]. In fact, such cognitive resilience effect receives attention in the entire neuropathology. Encountered with physical injury, some individuals maintain their cognitive levels by utilizing the physical or functional redundancy of the brain [11]. This has also been observed in Alzheimer's disease (AD) and age‐related dementia, yet the mechanism is not fully understood [11, 12]. Early researches focused on passive physical elements such as intracranial volume, which is summarized as the term “brain reserve” [13]. Conversely, the cognitive reserve is active, available through life experience, and has increasingly raised the interest of neurologists [14, 15]. However, current knowledge for cognitive reserve stays at education or leisure activity. It relies on self‐reported events rather than current status, thus hampering the understanding of how the brain preserves its function against various damage.

It is essential to know the detailed contribution to cognitive reserve from measurable and explainable factors. An entry point is brain network feature. As a hierarchical network system, human brain consists of modules, each of which owns individual capability and can work for complex tasks in combination [16, 17]. The topological organization of these modules is crucial for the function, cost, and evolution of brain [16, 18]. Segregation and integration are the major couple of concepts describing the functional organization [19]. Segregation means high density of intra‐module functional connections, implying independence of each module, whereas integration means frequent inter‐modular communications, implying the global synchronism. During resting state, the large‐scale network stays at a balance between being totally segregated or integrated. Yet, the equilibrium point varies among individuals [20, 21]. A recent study utilizing graph theory has reported that higher system segregation is associated with better cognitive resilience in Alzheimer's disease [22]. However, there still lacks of understanding the mechanism behind this phenomenon. Also, there lacks of report on this effect for vascular cognitive impairment. Therefore, whether and how resting‐network organization influence cognitive outcomes in cerebral CSVD deserves exploration.

Recently, a nested‐spectral partition (NSP) approach was introduced to detect the network hierarchical structure based on functional MRI signals [23]. This eigenmode‐based approach adopts the assumption that cooperatively activated regions should share the same eigenvector sign, while independently activated regions do not. Therefore, the low‐order (e.g., first level) eigenvalue characterize the extend of whole brain blood‐oxygen signal synchronization. The competition between low‐order and high‐order eigenmode, quantified by a hierarchical balance index, indicates the preference toward segregation or integration [20].

Our study aims to explore whether and how segregation‐integration preference would influence the cognitive change against brain lesion. CSVD serves as the source of brain damage, which is quantified by a composite z‐score based on several typical MRI findings. Applying the NSP method, the resting functional organization characterization are captured. General cognitive ability as well as five cognitive domains are included into analysis.

## 2 Materials and Methods

This single‐center study was approved by the Research Ethics Committee of our hospital. The request of written informed consent forms was waived due to the retrospective nature.

**FIGURE 1:**
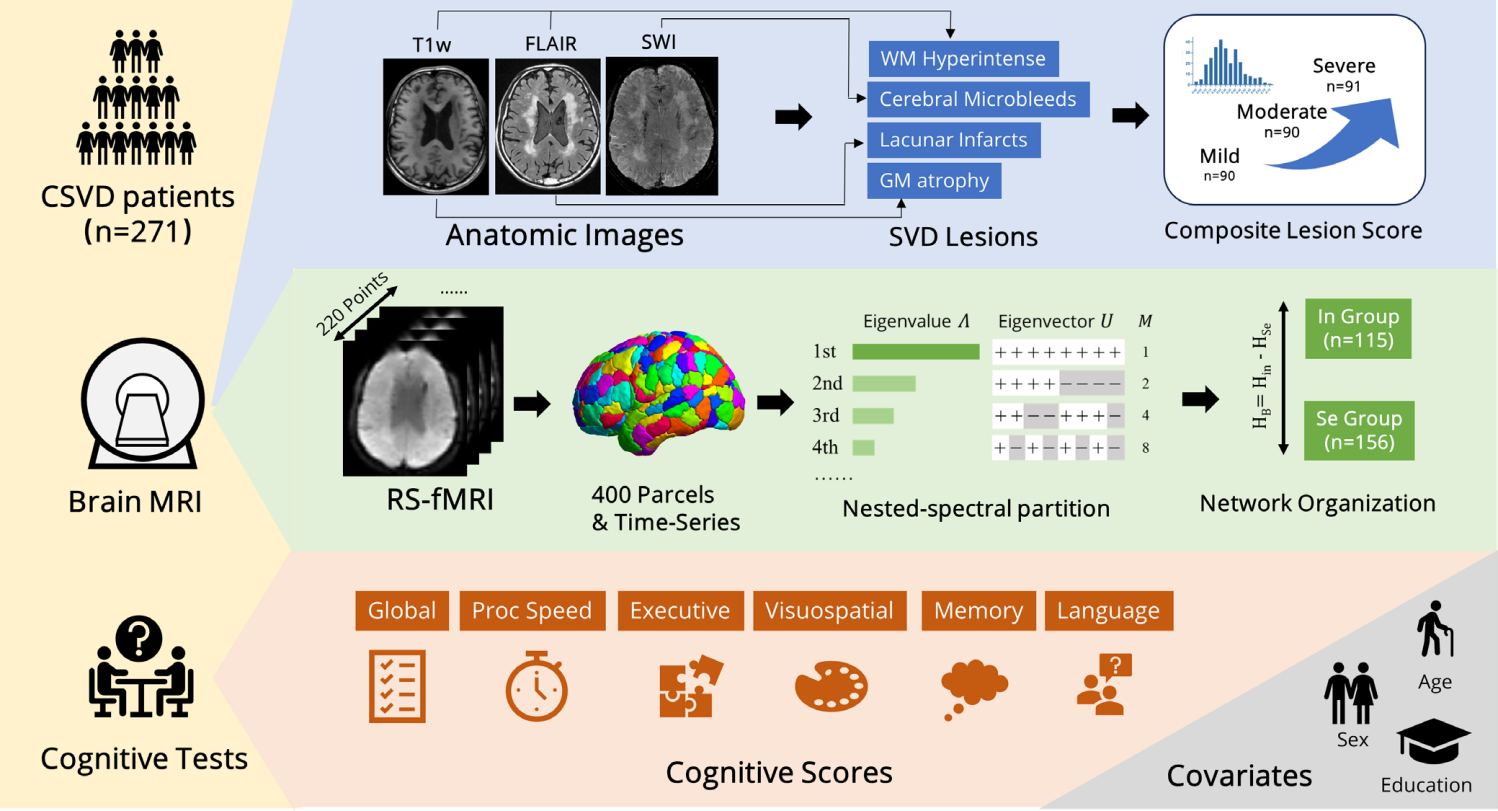
Diagram illustrating the main workflow of this study.

A diagram of workflow was given in Figure 1 which also illustrated our main study design. The following assumptions were adopted in this study.

1. Damage originated from CSVD are continuous, constructing a spectrum from minor imaging findings to severe ones. Therefore, we adopted composite lesion z‐score, and apply WMH volume instead of semi‐quantified scores like Fazekas score.
2. Cognitive functions are continuous status and may be hampered or preserved through different mechanisms of brain network. Therefore, we did not classify patients into NCI, MCI, or dementia but quantify the cognitive status using z‐scores for each domain.
3. Although aging serves as significant etiology for vascular damage, it was controlled as a covariate here since imaging findings were already assessed as CSVD lesion burden.

### 2.1 Participants

**FIGURE 2:**
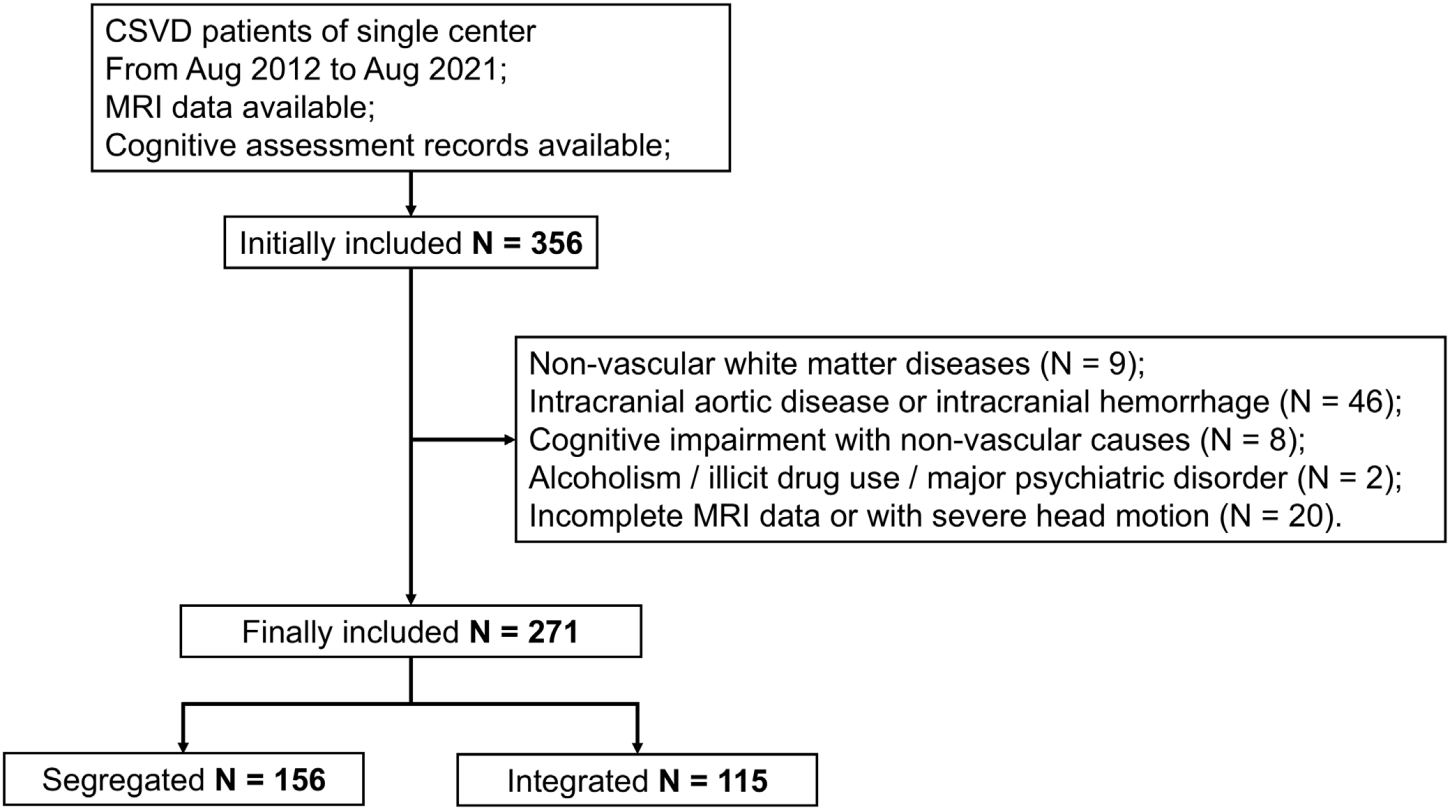
Flow diagram illustrating the participant inclusion and exclusion procedure.

Our study reviewed the data of consecutive patients with CSVD from August 2012 to August 2021. Inclusion criteria were as follows: (1) Patients diagnosed as CSVD, either with symptoms or by imaging examination; (2) MRI data available; (3) With records of the neuropsychological assessment at our hospital. Exclusion criteria were as follows: (1) non‐vascular white matter diseases (e.g., infection, malignancy) (2) Intracranial aortic disease or intracranial hemorrhage; (3) Cognitive impairment with non‐vascular causes (e.g., Alzheimer's disease, Parkinson's disease, or hydrocephalus); (4) alcoholism, illicit drug use disorder, or major psychiatric disorder; (5) Incomplete MRI data or with severe head motion. Finally, 271 CSVD patients were included in this study. Details of the patient inclusion and exclusion procedure is given in Figure 2. Demographic information including age, sex, and year of education were recorded. More than 9 years of education was defined as higher education.

### 2.2 MRI Acquisition

All subjects underwent MR examination at a 3.0T Scanner (Signa HDxt, GE Healthcare, USA) using an eight channel standard head coil. Foam paddings were applied inside the coil to reduce the head motion. Resting‐state functional MRI (rs‐fMRI), T1‐weighted (T1w) structural images, T2‐weighted fluid‐attenuated inversion recovery (FLAIR) images, and susceptibility‐weighted images (SWI) were acquired for each patient. Rs‐fMRI were acquired by gradient echo‐planar imaging (GE‐EPI) sequence with the following protocol: field of view (FOV) = 230 × 230 mm^2^, repetition time (TR)/echo time (TE) = 2000/24 ms, acquisition matrix = 64 × 64, flip angle (FA) = 90°, number of slices = 34, slice thickness/gap = 4/0 mm. Totally 220 volumes (duration time 440 s) were acquired for each scan. Isotropic T1w was acquired by a 3D fast spoiled gradient recalled echo (SPGR) sequence with the following protocol: FOV = 256 × 256 × 156 mm^3^, TR/TE = 5.6/1.8 ms, acquisition matrix = 256 × 256 × 156, FA = 15°, inversion time (TI) = 450 ms. T2w FLAIR was acquired with: FOV = 256 × 256 mm^2^, TR/TE = 9075/150 ms, acquisition matrix = 128 × 128, number of slices = 66, slice thickness/gap = 2/0 mm, TI = 2250 ms. SWI was acquired using 3D SPGR with: 16 TEs, TE_min_/ΔTE/TE_max_ = 3.2/2.42/39.5 ms, TR = 42.5 ms, FA = 12°, FOV = 220 × 220 × 132 mm^3^, acquisition matrix = 256 × 256 × 66.

### 2.3 Functional MRI Post‐Processing

The standardized pipeline embedded in the Data Processing Assistant for Resting‐State fMRI toolbox were adopted in our study for rs‐fMRI pre‐processing [24]. Specifically, the pipeline includes slice‐time correction, head motion correction, nuisance covariate regression, spatial normalization to the Montreal Neurological Institute (MNI) space, temporal filtering, and spatially smoothing. To better describe the hierarchical structure of brain functional network, a large‐scale parcellation atlas with 400 regions were applied using Schaefer 2018's approach [25]. Time series for each region was extracted by averaging signals of all voxels within each region at each time‐point, generating 400 arrays containing 220 time‐points for each individual. Time series by 300 regions and 500 regions of the same approach were also obtained for a consistency test. Static functional connectivity (FC) matrix was constructed based on the 400‐region time series. Specifically, the Pearson correlation coefficient between signal arrays of two regions (e.g., region *i* and *j*) was calculated pair‐by‐pair, and was defined as *F*_*ij*_ (=*F*_*ji*_). Diagonal value of the static FC matrix was naturally 1.

### 2.4 Assessment of Functional Network Organization

An eigenmode‐based NSP approach was applied to identify the network organization characteristics [23]. Eigenvectors *U* and eigenvalues Λ were decomposed from the FC matrix. The hierarchical modules $M_{i}\left(i=1,\cdots ,N\right)$ were defined based on the eigenvectors U [20, 23]. The modular size in each level was indicated as $m_{i}\left(i=1,\cdots ,N\right)$. For each level of hierarchical module, weighted module number $H_{i}$ was calculated to reflect the hierarchical segregated and integrated interactions.

$$H_{i}=\frac{\Lambda^{2}M_{i}\left(1-p_{i}\right)}{N}$$

where $p_{i}$ was a correction factor for heterogeneous module size:

$$p_{i}=\sum_{j=1}^{N}\frac{\left|m_{j}-\frac{N}{M_{i}}\right|}{N}$$

After that, the network integration and segregation components were calculated as follows:

$$H_{\text{In}}=\frac{H_{1}}{N},\text{and}\;H_{\mathrm{Se}}=\sum_{i=2}^{N}\frac{H_{i}}{N}$$

Considering the potential influence of fMRI acquisition duration on the calculation, a calibration process is applied for both integration and segregation components [20]. Finally, the measure of hierarchical balance index for each individual was calculated.

$$H_{B}=H_{\text{In}}-H_{\mathrm{Se}}$$

This index globally measures the distance of each individual from balanced organization. The individuals were classified into two groups: segregated (Se) and integrated (In), separately with negative and positive *H*_*B*_. A stable FC matrix was obtained for both groups, utilizing the long time series containing fMRI signals of all segregated or integrated patients.

### 2.5 Lesion Load Assessment

In our study, brain lesions were assessed on the MRI which is the established tool for CSVD diagnosis [3, 5]. Common imaging findings of brain lesion for CSVD patients include WMH, LI, CMB, and gray matter (GM) atrophy [4]. The regions of WMH were segmented based on T1w and FLAIR images using a cluster‐based pipeline, UBO detector [26]. The GM and non‐GM (white matter & cerebrospinal fluid) regions were segmented based on T1w using Statistical Parametric Mapping 8 (https://www.fil.ion.ucl.ac.uk/spm/), and GM volume fractions were calculated. The above automatic assessments were performed under the supervision of a radiologist. LIs and CMBs were manually counted on FLAIR and SWI.

In order to estimate the overall lesion load for each individual, a composite lesion z‐score was calculated including the following lesion load indicators: (1) WMH volume (z_V_WMH_); (2) Number of LI (z_N_LI_); (3) Number of CMB (z_N_CMB_); (4) GM volume fraction (z_VF_GM_, negative contribution). The original composite z‐score were log‐transformed in order to achieve a distribution more in line with normal distribution (see Figure S1). Specifically, the transformation formula was:

$$\text{Lesion score}=\log\;\left(\left(z\_\text{VWMH}+z\_\mathrm{NLI}+z\_\text{NCMB}-z\_\text{VFGM}\right)/4+2.328\right)$$

where the constant 2.328 is decided by keeping the lowest lesion score as 0.100 in our dataset. Three stages of CSVD lesion load—mild, moderate, and severe—were tri‐equivalently divided according to the lesion score. Finally, these stages contain 90, 90, and 91 subjects, respectively.

### 2.6 Neuropsychological Assessment

The neuropsychological assessments for subjects were conducted within 1–4 days (median: 1 day) after the MRI examination. All patients underwent a battery of multi‐domain cognitive tests. The global cognitive performance was evaluated using the Montreal Cognitive Assessment (MoCA) [27]. Normalized scores of five cognitive domains, including processing speed, executive, memory, visuospatial, and language capability, were assessed [28]. Processing speed was assessed by trail‐making test (TMT)‐A, Stroop color‐word test (Stroop)‐A, and Stroop‐B [29, 30]. Executive ability was assessed using TMT‐B, Stroop‐C, and digit symbol substitution test (DSST) [29, 30, 31]. Memory was assessed using auditory verbal learning test (AVLT), where performance without‐delay (AVLT 1 + 2 + 3), short‐delay free recall (AVLT 4), and long‐delay free recall (AVLT 5) measurements were regarded as three indicators [32]. Visual ability was assessed using Rey‐Osterrieth complex figure test (ROCFT) [33]. Language ability was assessed using Boston naming test (BNT) and category verbal fluency test (VFT) [34, 35].

### 2.7 Statistics

Statistical analysis was performed with SPSS, version 23.0 (IBM Corporation). Based on the multi‐scale atlas generation approach by Schaefer et al. [25], an inter‐scale consistency of 400‐parcel derived *H*_*B*_ to those by 300‐ and 500‐parcel was conducted using two‐way mixed intra‐class coefficient (ICC). Kolmogorov–Smirnov tests were applied for assessing normality. Comparisons of categorical variables between different groups were performed using Pearson's $\chi^{2}$ test. Continuous variables of normal distribution were compared using Student's *t* test. Continuous variables without normality were compared using Mann–Whitney *U* test.

A hierarchical regression analysis was applied to investigate the influence of *H*_*B*_ on cognitive functions. Specifically, after controlling age, sex, and education (block 1), a step‐wise regression model was constructed, sequentially using lesion score (block 2), *H*_*B*_ (block 3), and their interaction: lesion score × *H*_*B*_ (block 4). The improvement of *R*‐square (∆*R* ^2^) and the significance of *F* changes were obtained at each block. This hierarchical regression was repeated to assess the influence of education for a comparative purpose. Specifically, after controlling age and sex (block 1), a step‐wise regression model was constructed sequentially using lesion score (block 2), education (block 3), and their interaction: lesion score × education (block 4).

Within each stage of CSVD damage, group comparisons were conducted using two‐tailed *t* test for all cognitive scores between segregated and integrated individuals. A missing at random assumption was adopted for missing data (education level and cognitive scores), with pairwise deletion applied in analysis. A *p* value of < 0.05 was considered statistically significant for all the tests.

## 3 Results

### 3.1 Distribution of Segregation‐Integration Preference by NSP

Table 1 lists the demographic information of participants included in this study. The distribution of the log‐transferred lesion score could been seen in Figure S1b. The dividing threshold was 0.230 between mild (*n* = 90) and moderate damage (*n* = 90), and 0.395 between moderate and severe damage (*n* = 91).

**TABLE 1:** Demographic information of CSVD patients included in the study.

	All patients (*N* = 271)	Mild damage (*N* = 90)	Moderate damage (*N* = 90)	Severe damage (*N* = 91)
Age (year)	66.6 ± 7.8	64.3 ± 7.7	66.2 ± 7.8	69.3 ± 7.4
Sex				
Male	211 (78)	77 (86)	68 (76)	66 (73)
Female	60 (22)	13 (14)	22 (24)	25 (27)
Education (year)	10.3 ± 3.2	10.7 ± 3.4	10.2 ± 3.4	10.0 ± 2.7
Lesion				
WMH volume (cm^3^)	17.4 ± 16.0	4.8 ± 3.1	13.1 ± 7.2	34.4 ± 15.5
CMB number	3.4 ± 6.9	0.5 ± 1.1	1.9 ± 2.3	8.0 ± 10.3
LI number	2.7 ± 3.8	0.7 ± 0.8	1.8 ± 1.8	5.8 ± 4.9
GM Volume (%)	36.5 ± 3.9	39.9 ± 2.3	36.4 ± 2.2	32.9 ± 3.3
MoCA	22.7 ± 4.5	24.7 ± 3.6	22.7 ± 3.9	20.5 ± 5.1

*Note:* The values are given in mean ± SD or number (percentage).

Abbreviations: CMB, cerebral micro‐bleeding; GM, gray matter; LI, lacunar infarct; MoCA, Montreal cognitive assessment; SD, standard deviation; WMH, white matter hyperintense.

**FIGURE 3:**
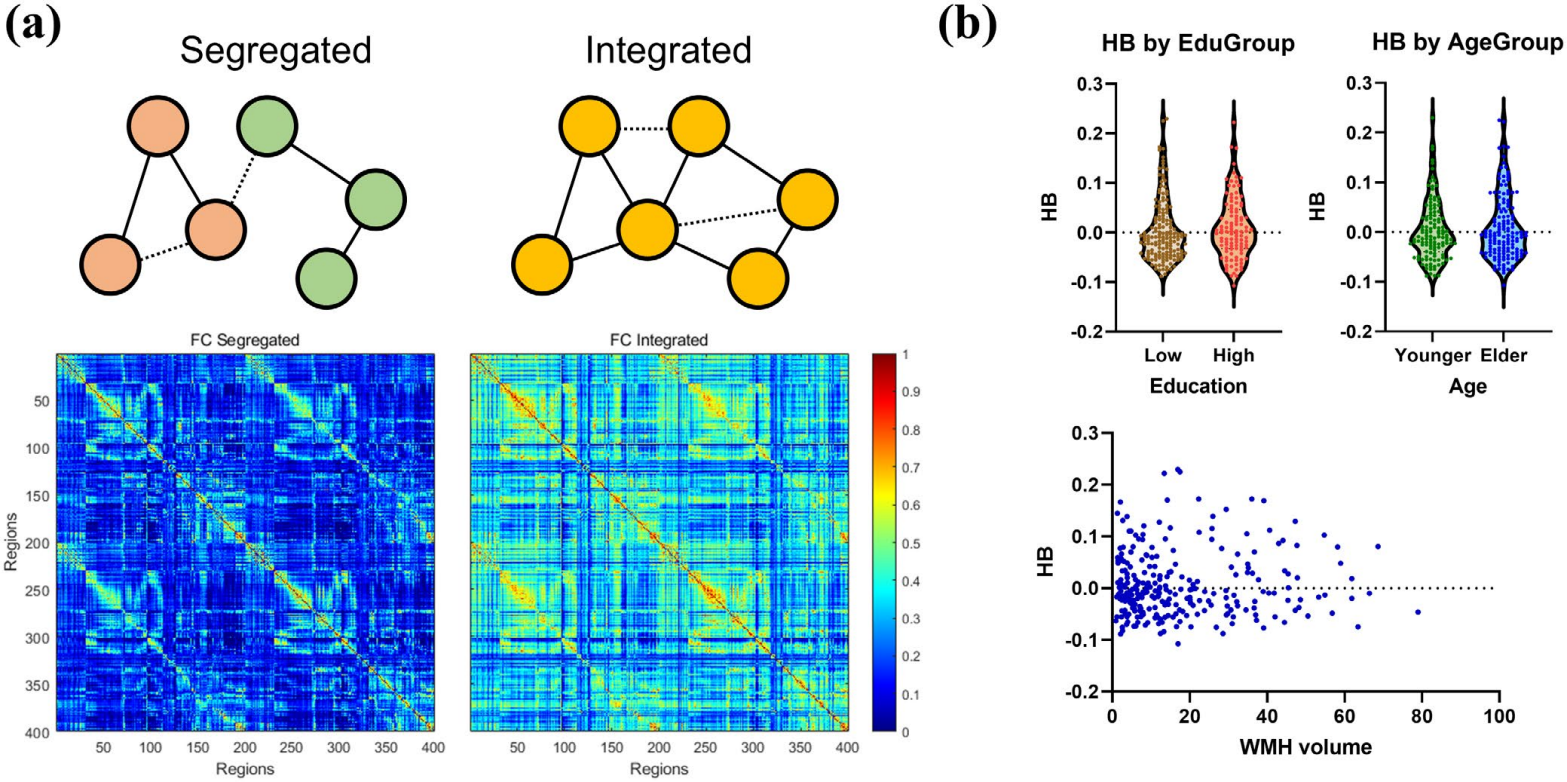
Illustration of functional segregation and integration. (a) Stable functional connectivity matrices by 156 SVD patients with segregated network, and by 115 patients with integrated network. Higher overall connectivity could be observed for the integrated group. (b) Distribution of *H*_*B*_ among CSVD patients. Group comparison indicated no significant difference for *H*_*B*_ among age and education groups. Distribution of *H*_*B*_ does not show obvious alteration toward increasing WMH volume.

There were 156 CSVD patients classified into segregated (*H*_*B*_ < 0) and 115 into integrated group (*H*_*B*_ > 0). Stable FC matrix by 156 CSVD patients with segregated network demonstrated larger connectivity than that by 115 patients with integrated network (Figure 3a). The integrated stable FC turned out larger first‐order eigenvalue compared to that for segregated one (147.4 vs. 80.5), as expected. The consistency of *H*_*B*_ among different network scale is good (Figure S2), with ICC 0.990 to 300‐parcel and 0.996 to 500‐parcel.

Among clinical characteristics, only age displayed normality. Among cognitive scores, executive and memory scores displayed normality, while the others did not. Table 2 presents the results for comparison of clinical characteristics between segregated and integrated individuals. No significant difference was found between age, sex, and education between segregated and integrated groups (all *p* > 0.05), indicating independence for resting‐network organization from these factors. Moreover, no difference was found for global lesion score or any specific damages (WMH, CMBs, Lis, and gray matter atrophy, all *p* > 0.05). The distribution of *H*_*B*_ between binary age and education groups, and crossing the progression of WMH are visualized in Figure 3b.

**TABLE 2:** Comparison of clinical characteristics between segregated and integrated group.

	Segregated group (*N* = 156)	Integrated group (*N* = 115)	*p*
Age (year)	67.2 ± 8.0	65.8 ± 7.6	0.411^a^
Sex			
Male	116 (74)	95 (82)	0.142^b^
Female	40 (26)	20 (18)	
Education (year)	10.1 ± 3.3	10.6 ± 2.9	0.079^c^
WMH volume (cm^3^)	17.6 ± 15.7	17.2 ± 16.3	0.462^c^
CMB number	3.2 ± 6.0	3.8 ± 8.1	0.522^c^
LI number	2.7 ± 3.9	2.8 ± 3.7	0.266^c^
Gray matter volume (%)	36.2 ± 3.9	36.8 ± 3.9	0.106^a^
Log‐transferred lesion score	0.310 ± 0.144	0.302 ± 0.154	0.346^c^

*Note:* The values are given in mean ± SD or number (percentage).

Abbreviations: CMB, cerebral micro‐bleeding; LI, lacunar infarct; WMH, white matter hyperintense.

^a^ Student's *t* test.

^b^ Chi‐square test.

^c^ Mann–Whitney *U* test.

### 3.2 Interaction Effects Between Lesion Load and Hierarchical Balance Index

**FIGURE 4:**
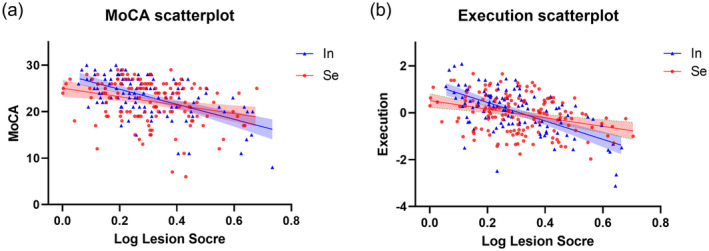
Scatterplot of (a) global cognitive score (MoCA) and (b) execution z‐score versus log‐transformed lesion score. Data of segregated and integrated resting‐network are separately notated. The regression lines (with 95% confidence interval) were given, displaying the interaction effects between lesion, and resting‐state network organization.

The results of hierarchical regression analysis are given in Table 3. After controlling age, sex, and education (block 1), log‐transferred lesion score demonstrated negative contribution to global cognition as well as all cognitive domains (*β* = −0.22~−0.35, ∆*R* ^2^ = 0.046~0.112, all *p* < 0.001). The inclusion of *H*_*B*_ (block 3) did not show significant contribution to the model in all these domains, although marginal significance was found in memory (*β* = 0.10, ∆*R* ^2^ = 0.011, *p* = 0.069). However, interaction between *H*_*B*_ and lesion score (block 4) was significantly associated with MoCA (*β* = −0.27, ∆*R* ^2^ = 0.013, *p* = 0.034) as well as execution score (*β* = −0.34, ∆*R* ^2^ = 0.023, *p* = 0.002). The interaction effects could be directly observed by the scatterplots (Figure 4) showing cross‐over between fitting lines of segregated and integrated group. Specifically, segregated individuals showed lower initial cognitive score but slower decreasing toward severer CSVD, both for global and executive ability.

**TABLE 3:** Hierarchical regression analysis for lesion load and resting‐network organization on cognitive performances, controlling age, sex, and education.

	MoCA			Processing speed			Execution		
	*β*	∆*R* ^2^	*p*	*β*	∆*R* ^2^	*p*	*β*	∆*R* ^2^	*p*
Age, sex, education	−0.25, 0.04, 0.24	0.121	< 0.001***	−0.28, 0.04, 0.22	0.111	< 0.001***	−0.35, 0.03, 0.32	0.221	< 0.001***
LLS	−0.31	0.088	< 0.001***	−0.31	0.092	< 0.001***	−0.35	0.112	< 0.001***
*H*_*B*_	0.04	0.002	0.455	0.04	0.003	0.353	0.01	< 0.001	0.929
LLS × *H*_*B*_	−0.27	0.013	0.034*	−0.05	< 0.001	0.978	−0.34	0.023	0.002**

	Memory			Visuospatial			Language		
	*β*	∆*R* ^2^	*p*	*β*	∆*R* ^2^	*p*	*β*	∆*R* ^2^	*p*
Age, sex, education	−0.29, 0.06, 0.20	0.110	< 0.001***	−0.10, 0.14, 0.14	0.056	< 0.001***	−0.15, 0.08, 0.34	0.154	< 0.001***
LLS	−0.29	0.075	< 0.001***	−0.29	0.078	< 0.001***	−0.22	0.046	< 0.001***
*H*_*B*_	0.10	0.011	0.069	0.06	0.003	0.323	0.08	0.006	0.175
LLS × *H*_*B*_	0.01	< 0.001	0.978	−0.04	< 0.001	0.791	−0.13	0.003	0.317

Abbreviations: LLS, log‐transformed lesion score; MoCA, Montreal cognitive assessment. SIgnificance symbols: * *P* < 0.05; ** *P* < 0.01; *** *P* < 0.001.

A comparative analysis on education, instead of *H*_*B*_, was given in Figure S3 and Table S1. Higher education could maintain higher cognitive scores for both global (*β* = 0.21, ∆*R* ^2^ = 0.041, *p* < 0.001) and executive function (*β* = 0.29, ∆*R* ^2^ = 0.081, *p* < 0.001). Nevertheless, its interaction to lesion score has no significant contribution. Notably, no cross‐over can be observed between the fitting lines in executive function.

### 3.3 Initial Reserve Effects by Integration in Several Cognitive Abilities

**FIGURE 5:**
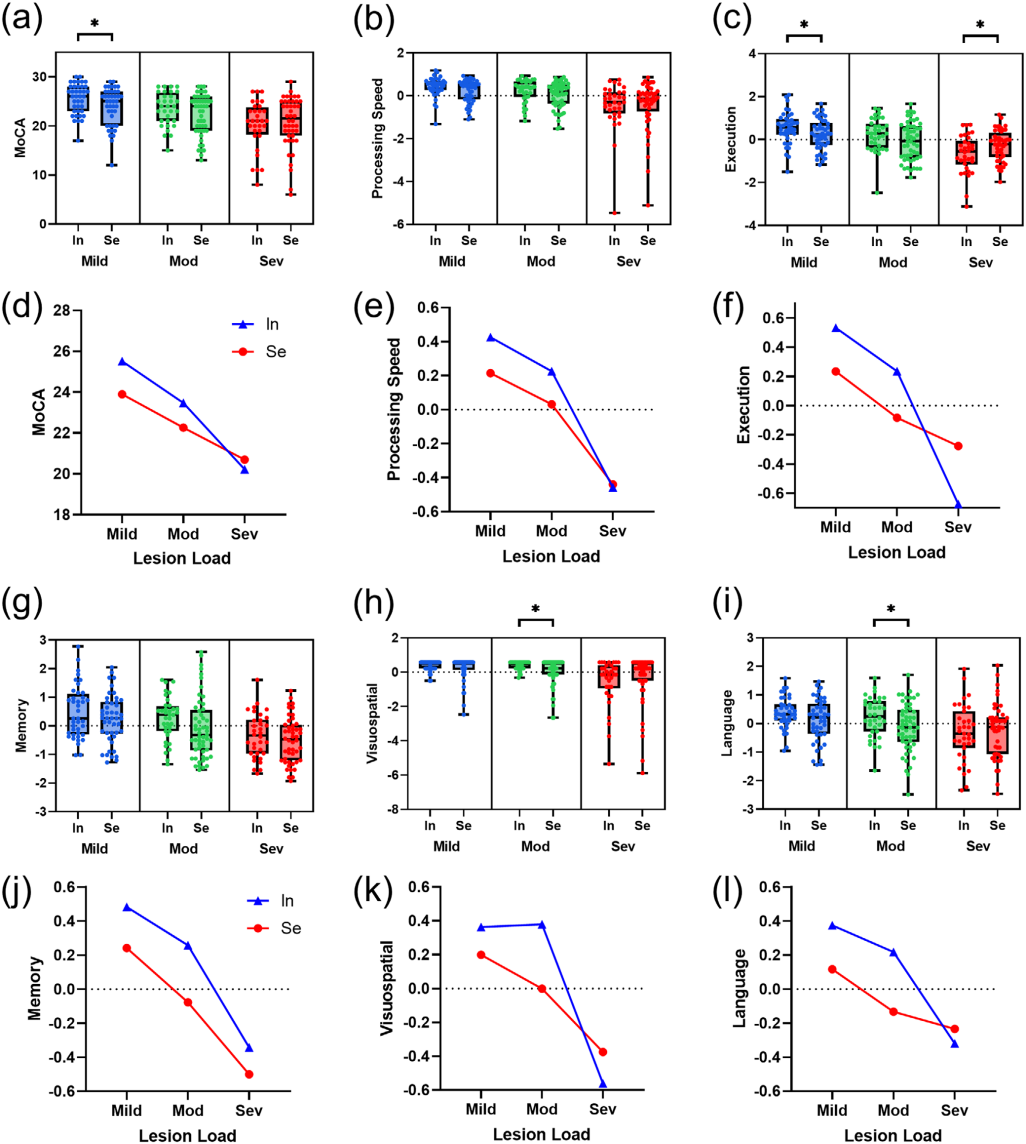
During the accumulation of CSVD damage, segregated and integrated patients display distinct cognitive impairment features at specific stages. (a–c) and (g–i) Nested boxplots showing the comparison of cognitive scores between the two resting‐network group in each stage. (d–f) and (j–l) Cognitive decreasing curves using the average value of the cognitive scores.

Stage‐based analysis for the cognitive scores between the two resting‐network groups were given (Figure 5), illustrating results from sub‐dataset of mild, moderate, and severe lesion load separately. For cohort with mild lesion load, segregated patients exhibit significant lower cognitive score than integrated ones in global (23.9 ± 3.9 vs. 25.5 ± 3.1, *p* = 0.044) and execution (0.235 ± 0.678 vs. 0.535 ± 0.688, *p* = 0.049). Marginal significance exists in processing speed (0.215 ± 0.530 vs. 0.426 ± 0.452, *p* = 0.089) and language (0.117 ± 0.751 vs. 0.375 ± 0.545, *p* = 0.069). In the moderate lesion stage, segregated patients show lower cognitive score in visuospatial (−0.001 ± 0.804 vs. 0.379 ± 0.249, *p* = 0.034) and language ability (−0.133 ± 0.849 vs. 0.218 ± 0.704, *p* = 0.037), as well as lower execution (−0.083 ± 0.888 vs. 0.236 ± 0.763, *p* = 0.074) and memory (−0.077 ± 1.067 vs. 0.258 ± 0.753, *p* = 0.087) yet with marginal significance. A cross‐over of cognitive scores could be observed toward severe lesion stage in global, execution, visuospatial, and language domain (Figure 4). Accordingly, significant higher score for segregated patients was found in execution (−0.277 ± 0.717 vs. −0.675 ± 0.883, *p* = 0.027).

## 4 Discussion

Across the CSVD patients with various lesion stages in this study, we found that resting‐state functional organization, quantified by the NSP method, could impact the way cognitive level decreases. In addition, we found main and interaction effects for modular index and lesion load on both global and executive cognitive scores, independent from age and education level. Moreover, higher scores for integrated individuals presented in mild or moderate lesion stage for global, processing speed, executive, visuospatial, and language ability.

Our findings suggest that resting‐state network organization configures CSVD‐induced cognitive decline. Moreover, this procedure varies among stages of damage, leading to notable interaction effects. Specifically, the cognition cross‐over between the two groups exists during the lesion load evolving from moderate to severe. Integrated individuals are less susceptible to cognitive decline at early‐stage damage. Such advantage manifested as significant initial‐stage difference in global cognitive and executive ability. Nevertheless, as the disease progresses, segregation resists further decline, while integration accelerates it. Eventually, in segregated patients with severe CSVD damage, some cognitive functions are, reversely, better than integrated patients, although only executive function showed significant difference.

Generally, the integrated individuals have a higher cognitive level at early stage, indicating a cognitive resilience effect for CSVD patients. The benefit brought by integration is more consistent with an initial advantage model, but with a higher “workload” [36]. This observation is fresh, different from previous models on conventional factors like education or leisure activities on CSVD [10], or those in AD and multiple sclerosis [12, 37]. Our comparative analysis on education also reveals distinct characteristics, that interaction effect has a weak or even none contribution. The biological and neurophysiological mechanisms that underlie cognitive reserve are not fully understood [13]. While education, occupation, and leisure activities are common proxies, they are more like a source rather than a direct brain property. Our findings propose that functional network preference would contribute to the cognitive reserve in CSVD. The balanced distribution among demographic characteristics suggests that this preference is independent from age, sex, education, or lesion load. Although a previous research suggests larger system segregation develops toward elder volunteers, the trend is less pronounced in individuals over age of 60, who constitute the primary risk group for cerebral disease [21].

Our findings offer insights into the mechanism behind vascular cognitive impairment. The integration‐segregation pair shares converse advantages and disadvantages, reaching a balance between them in human brain network [18, 20]. Integrated network shows larger global signal synchronization than segregated network, indicating more inter‐community connections but less intra‐community ones. Demyelination and axonal loss, which are common CSVD pathologies, could lead to disconnection of signal pathways for both inter‐ and intra‐community ones [2]. In early stage of damage, integrated resting‐network compensates for the partial deconstruction by calling other unaffected pathways. Yet, this “connection reserve” fails under severe damage. Conversely, segregated individuals own their advantages for better utilization of the regional subsystems, which adopts physically shorter intra‐community connections. These pathways could survive even under severe damage.

Recent meta‐analysis suggests that CSVD‐induced cognitive impairments take place globally, affecting cognitive domains including processing speed, executive, memory, visuospatial, and language ability [7]. Our findings verified that lesion load is a significant factor in all domains. Among these domains, processing speed and executive function are two primary ones affected by CSVD [38]. Integrated network preserved both of them at early‐stage lesion, evidenced by the significant inter‐group differences. Executive function refers to complex task involving cooperation among several sub‐functions, for example, attention, vision, judgment, and motion. It is most sensitive to the proposed reserve mechanism, turning out to have the largest *R* ^2^ improvement among the cognitive domains.

CSVD and AD shares similarity in cognitive outcome but differs in pathology. A recent study on AD reported slower cognitive decline toward pathology accumulation for segregated individuals [22]. Yet different from our findings in CSVD, they proposed no initial advantage for integration. The pathologies of AD mainly include amyloid and tau deposition, affecting medial temporal lobe. The distinct role of resting‐network organization implies that AD pathologies destroy less inter‐community connections than vascular ones. Notably, memory is the domain to be most affected by AD. It also showed a distinct feature of cognitive decreasing curve, where integrated group gains a slightly higher (not significant) score in all three stages. The lack of end‐stage cross‐over indicates that memory may requires special functional connections. Previous research proposed that memory is not influenced in proportion to execution function by vascular lesions [28].

Our study does have some limitations. Technically, the hierarchical modular calculated from eigenmode‐based method would be affected by the time duration of the fMRI scanning, especially for the segregation components [20]. Although a calibration has been taken, the relatively short acquisition duration (440 s) may still hinder an accurate measurement of *H*_*B*_. The sample size for patients suffering severe cognitive decrease (e.g., dementia) is limited due to the difficulty in complete cognitive tests and fMRI examination. The measurement of visuospatial function is restricted by the fact that only single cognitive assessment, ROCFT, was applied. Accordingly, a slightly raised z‐score in integrated group from mild to moderate lesion stage was observed (Figure 5, panel k), probably associated with this limitation.

In conclusion, resting‐state functional organization preference to segregation or integration would configure the cognitive decline against cerebral small vessel disease. Generally, integrated individuals show better cognitive functions in mild and moderate but not in severe damage stage compared to segregated ones, and a cognitive cross‐over exists for most domains. These findings offer insights into how CSVD lead to cognitive impairment, as well as the underlying mechanism of cognitive reserve.

## Author Contributions

**Wentao Hu:** conceptualization, methodology, formal analysis, visualization, writing – original draft. **Yao Wang:** conceptualization, investigation, data curation, writing – original draft. **Zhenhui Xie:** formal analysis, visualization. **Mianxin Liu:** methodology, investigation. **Xu Han:** data curation, formal analysis. **Ying Hu:** validation. **Xingrui Wang:** data curation. **Yongming Dai:** writing – review and editing. **Qun Xu:** funding acquisition, resource, project administration. **Yan Zhou:** project administration, funding acquisition, writing – review and editing.

## Conflicts of Interest

The authors declare no conflicts of interest.

## Supporting information


Data S1.

## Data Availability

Original data included in this study are mainly human MR images and clinical data acquired at our hospital, and is administrated according to the clinical and research ethics policies of the hospital. Therefore, the data could be not be acquired from a public repository, but are available from the corresponding author on reasonable and formal requests.
