# Supplementary material for: Functional Segregation‐Integration Preference Configures the Cognitive Decline Against Cerebral Small Vessel Disease: An MRI Study
Source: CNS Neurosci Ther. 2024 Dec 17;30(12):e70162. doi: 10.1111/cns.70162 (PMC11652672; doi:10.1111/cns.70162)
Supplement: Supplementary file 1 — Data S1. [file CNS-30-e70162-s001.docx]

**Supplementary Materials**


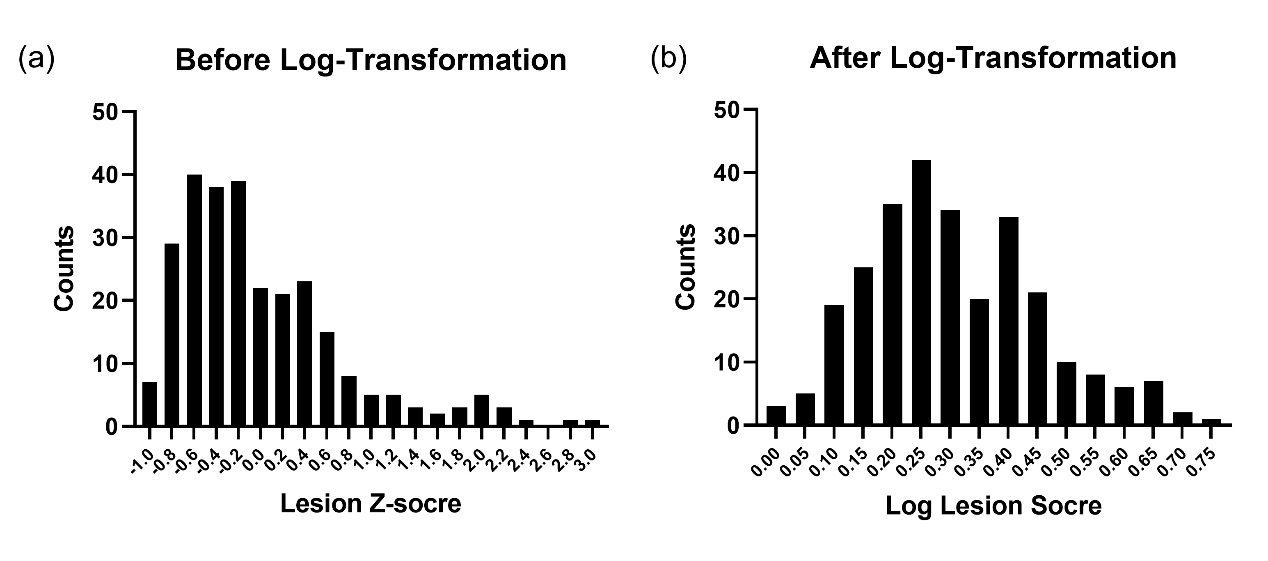


**Figure S1** Histogram of lesion score (a) before log-transformation (original composite z-score); (b) after log-transformation. The log-transformed lesion score is more in line with normal distribution and adopted in further analysis.


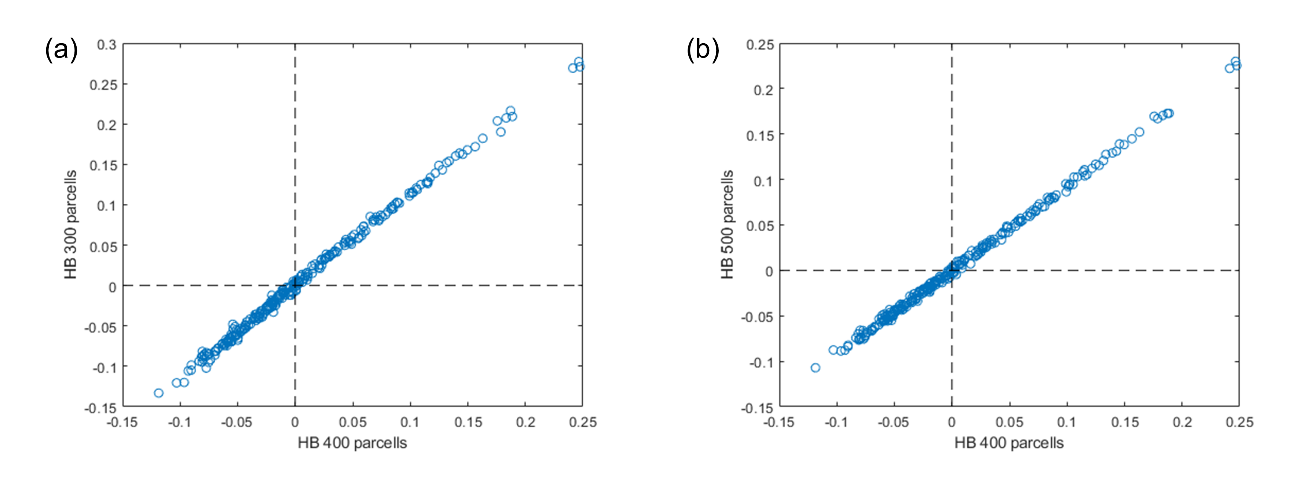


**Figure S2** Hierarchical balance index for each individual is stable through different choice of parcel number. (a) Schaefer’s 300 versus 400 parcels; (b) Schaefer’s 400 versus 500 parcels.


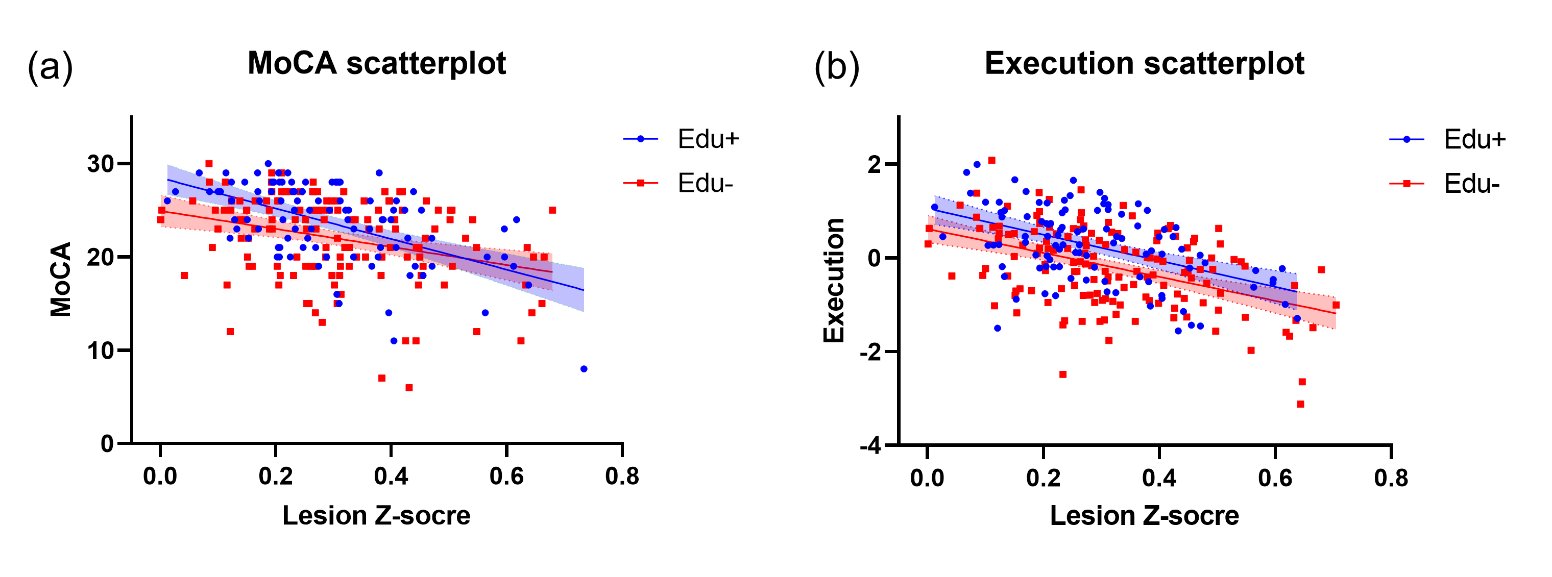


**Figure S3** Scatterplot of (a) general cognitive score MoCA and (b) execution z-score versus log-transformed lesion score. Data of higher (Edu+) and lower education (Edu-) individuals are separately notated. The regression lines (with 95% confidence interval) were given.

**Table S1** Hierarchical regression analysis for lesion load and education on cognitive performances, controlling age and sex. Interaction between lesion and education do not significantly contribute to the model.

|  | MoCA | | | Processing Speed | | |
| --- | --- | --- | --- | --- | --- | --- |
|  | β | ∆R^2^ | *P*-value | β | ∆R^2^ | *P*-value |
| Age, Sex | -0.24, 0.08 | 0.067 | <0.001 *** | -0.34, 0.06 | 0.120 | <0.001 *** |
| LLS | -0.33 | 0.101 | <0.001 *** | -0.38 | 0.132 | <0.001 *** |
| Education | 0.21 | 0.041 | <0.001 *** | 0.29 | 0.081 | <0.001 *** |
| LLS $\boldsymbol{\times}$ Edu | -0.24 | 0.004 | 0.234 | -0.07 | <0.001 | 0.735 |

Note:

LLS – Log-transformed lesion score; MoCA = Montreal cognitive assessment
